# Blood transfusion improves renal oxygenation and renal function in sepsis-induced acute kidney injury in rats

**DOI:** 10.1186/s13054-016-1581-1

**Published:** 2016-12-20

**Authors:** Lara Zafrani, Bulent Ergin, Aysegul Kapucu, Can Ince

**Affiliations:** 1 Department of Translational Physiology, Academic Medical Center, Amsterdam, The Netherlands; 2 Department of Intensive Care, Erasmus MC, University of Medical Center, Rotterdam, The Netherlands; 3 Department of Biology, Faculty of Science, University of Istanbul, Istanbul, Turkey

**Keywords:** Acute kidney injury, Sepsis, Microcirculation, Blood transfusion, Renal oxygenation, Endothelial dysfunction

## Abstract

**Background:**

The effects of blood transfusion on renal microcirculation during sepsis are unknown. This study aimed to investigate the effect of blood transfusion on renal microvascular oxygenation and renal function during sepsis-induced acute kidney injury.

**Methods:**

Twenty-seven Wistar albino rats were randomized into four groups: a sham group (*n* = 6), a lipopolysaccharide (LPS) group (*n* = 7), a LPS group that received fluid resuscitation (*n* = 7), and a LPS group that received blood transfusion (*n* = 7). The mean arterial blood pressure, renal blood flow, and renal microvascular oxygenation within the kidney cortex were recorded. Acute kidney injury was assessed using the serum creatinine levels, metabolic cost, and histopathological lesions. Nitrosative stress (expression of endothelial (eNOS) and inducible nitric oxide synthase (iNOS)) within the kidney was assessed by immunohistochemistry. Hemoglobin levels, pH, serum lactate levels, and liver enzymes were measured.

**Results:**

Fluid resuscitation and blood transfusion both significantly improved the mean arterial pressure and renal blood flow after LPS infusion. Renal microvascular oxygenation, serum creatinine levels, and tubular damage significantly improved in the LPS group that received blood transfusion compared to the group that received fluids. Moreover, the renal expression of eNOS was markedly suppressed under endotoxin challenge. Blood transfusion, but not fluid resuscitation, was able to restore the renal expression of eNOS. However, there were no significant differences in lactic acidosis or liver function between the two groups.

**Conclusions:**

Blood transfusion significantly improved renal function in endotoxemic rats. The specific beneficial effect of blood transfusion on the kidney could have been mediated in part by the improvements in renal microvascular oxygenation and sepsis-induced endothelial dysfunction via the restoration of eNOS expression within the kidney.

**Electronic supplementary material:**

The online version of this article (doi:10.1186/s13054-016-1581-1) contains supplementary material, which is available to authorized users.

## Background

Acute kidney injury (AKI) is a serious complication of sepsis in the intensive care setting that is associated with increases in the likelihood of death, prolonged hospital stays, and increased costs of care [1, 2]. The balance between renal microcirculatory oxygen delivery and cellular oxygen consumption is significantly disturbed during sepsis. Indeed, renal microvascular oxygenation is highly sensitive to endotoxemia, and renal hypoxia plays a crucial role in the pathogenesis of sepsis-induced AKI [3]. Moreover, the negative effects of anemia in critically ill patients with AKI have been previously described, and a hemoglobin concentration below 9 g/dl upon intensive care unit admission has been found to be an independent risk factor for mortality [4]. Previous studies that have measured renal microcirculatory oxygen pressure in experimental models of shock have consistently demonstrated that while fluid resuscitation (FR) is able to correct systemic hemodynamic variables it is unable to correct renal microcirculatory dysfunction [5, 6]. Based on this inability of fluids to improve oxygen delivery to the renal microcirculation, we hypothesized that the transfusion of oxygen-carrying red blood cells would improve sepsis-induced hypoxia. Two clinical studies have examined the ability of blood transfusion (BT) to correct the perfusion and oxygenation of the microcirculation in septic shock patients [7, 8]. These studies demonstrated that the microcirculation is globally unaltered by BT in septic patients; however, patients with altered capillary perfusion at baseline improve after BT [7]. To date, no study has focused on the effect of BT on sepsis-induced AKI.

The purpose of this study was to investigate the effect of BT on renal microvascular oxygenation and renal outcome (e.g., renal function and structural changes) during sepsis-induced AKI. We used an endotoxemic model to examine the questions: (1) whether BT can correct sepsis-induced renal microcirculatory hypoxemia; (2) whether BT is superior to FR in terms of restoring renal hypoxemia during sepsis; and (3) whether an improvement in renal hypoxemia translates into a better renal outcome. We compared the effects of FR involving a crystalloid solution and BT on renal hemodynamic parameters, renal oxygenation, renal function, renal damage, and nitrosative stress to investigate the extent to which improvements could be achieved by the administration of an oxygen-carrying resuscitation fluid.

## Methods

### Animals

The experiments were performed on Wistar albino rats (Harlan Netherlands BV, Horst, The Netherlands) with a mean body weight of 325 ± 19 g.

### Experimental protocol

Surgical preparation and blood gas measurements are detailed in Additional file 1.

**Fig. 1:**
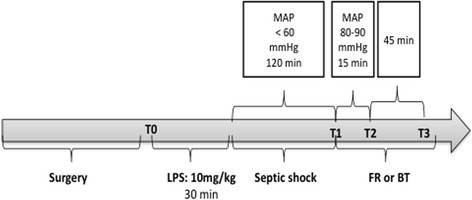
Experimental protocol. Time frame of the study. *BT* blood transfusion, *FR* fluid resuscitation, *LPS* lipopolysaccharide, *MAP* mean arterial pressure

The rats were randomized into four groups: (1) a sham operation group (control, *n* = 6), (2) a lipopolysaccharide (LPS) group (*n* = 7), (3) a LPS group with fluid resuscitation and a targeted mean arterial pressure (MAP) of 80–90 mmHg (LPS + FR, *n* = 7), and (4) a LPS group with blood transfusion and a targeted MAP of 80–90 mmHg (LPS + BT, *n* = 7). In the septic groups, a 30-min infusion of LPS (10 mg/kg; serotype 0127:B8; Sigma, The Netherlands) was given to induce septic shock. Fluid resuscitation was performed with a crystalloid solution (Ringer’s lactate; Baxter). Fluid resuscitation (median 4.64 ml, interquartile range 3–7 ml) and blood transfusion (median 4.19 ml, interquartile range 3–5.5 ml) (*p* = 0.2) were initiated 120 min after the end of LPS infusion and ended 15 min later. The experiment was ended 180 min after the end of LPS infusion or at the corresponding time point for the control group (Fig. 1). Of note, estimated blood volume in Wistar rats with a mean body weight of 325 g is 20.3 ml (from the equation of Lee and Blaufox [9]). Thus, 4.64 ml represents 22.8% of volemia and 4.19 ml represents 20.6% of volemia.

### Blood transfusion

Blood samples were obtained from donor inbred Wistar albino rats. The donor rats were anesthetized and mechanically ventilated. The right femoral artery was cannulated to draw blood samples. The blood samples were mixed with a standard citrate-phosphate-dextrose-adenosine anticoagulant solution at a 4:1 volume ratio. Allogeneic BTs from these donor rats were administered to the study group through the right jugular vein with a targeted MAP between 80 and 90 mmHg.

### Measurement of renal microvascular oxygenation

The renal microvascular partial pressure of oxygen (CμPO_2_) within the kidney cortex was measured by oxygen-dependent quenching of palladium-porphyrin phosphorescence using a phosphorimeter with a gated photomultiplier as previously described [10, 11]. Briefly, intravenously infused palladium-porphyrin binds to albumin. If excited by a flash of light (wavelength 530 nm), the palladium-porphyrin albumin complex emits phosphorescence (wavelength >700 nm). Depending on the oxygen concentration, the phosphorescence intensity decreases, and the relationship between the measured decay time and the partial pressure of oxygen (PO_2_) can be estimated using the Stern-Volmer relation [11].

### Measurement of renal blood flow

During the experimental procedure, the renal vessels were carefully separated with preservation of the nerves and adrenal gland. A perivascular ultrasonic transient time flow probe was placed around the left renal artery (type 0.7 RB; Transonic Systems Inc., Ithaca, NY, USA) and connected to a flow meter (T206; Transonic Systems Inc.) to continuously measure the renal blood flow.

### Calculation of derivatives of oxygenation parameters and renal vascular resistance

Renal oxygen delivery was calculated as follows: DO_2ren_ (ml/min) = renal blood flow × arterial oxygen content (1.31 × hemoglobin × arterial oxygen saturation) + (0.003 × arterial pressure of oxygen). Renal oxygen consumption was calculated as follows: VO_2ren_ (ml/min/g) = renal blood flow × (CaO_2_ – CvO_2_), where renal venous oxygen content (CvO_2_) is calculated as (1.31 × hemoglobin × renal venous oxygen saturation) + (0.003 × renal venous partial pressure of oxygen (rvPO_2_)) [12]. An estimation of the renal vascular resistance was made as follows: renal vascular resistance (dynes·sec·cm^–5^) = (MAP/renal blood flow) × 100 [13].

### Creatinine clearance

Creatinine clearance (ml/min) was assessed as an index of the glomerular filtration rate. Clearance was calculated using the following formula: Creatinine clearance = (U_crea_ × V)/P_crea_, where U_crea_ was the concentration of creatinine in urine, V was the urine volume per unit time, and P_crea_ was the concentration of creatinine in plasma.

### Renal energy efficiency for sodium transport: metabolic cost

The renal energy efficiency for sodium transport (VO_2_/TNa) was assessed using a ratio that was calculated from the total amount of VO_2_ over the total amount of sodium reabsorbed (TNa, mmol/min) according to the follow equation: ((U_crea_ × V)/P_crea_ × PNa) – UNa × V, where U_crea_ is the concentration of creatinine in the urine, V the urine volume per unit time, P_crea_ is the concentration of creatinine in the plasma, PNa is the concentration of sodium in plasma, and UNa is the concentration of sodium in the urine [14].

### Histological analysis

Four percent formalin-fixed, paraffin-embedded renal tissue sections (4 μm) were stained with periodic acid-Schiff + hematoxylin-eosin reagent. Histological changes in the cortex were assessed via quantitative measurements of tissue damage. The histological criteria for renal damage were tubular epithelial swelling, brush border loss, vacuolar degeneration, necrotic tubules, luminal cast formation, and invagination. The degree of kidney damage was estimated at 400× magnification using 10 randomly selected fields from each animal according to the following criteria: 0, normal; 1, areas of damage <10% of the tubules; 2, damage involving 10% to 25% of the tubules; 3, damage involving 25% to 50% of the tubules; and 4, damage in more than 50% of the tubules.

### Immunohistochemical analysis

Kidney sections (4 μm) were deparaffinized with xylene and rehydrated with decreasing percentages of ethanol and finally with water. Antigen retrieval was accomplished by microwaving the slides in citrate buffer (pH 6.0, Thermo Scientific, AP-9003-500) for 10 min. The slides were left to cool for 20 min at room temperature and then rinsed with distilled water. The endogenous peroxidase activity was blocked with 3% H_2_O_2_ for 10 min at room temperature, and the slides were later rinsed with distilled water and phosphate-buffered saline (PBS). Blocking reagent (Lab Vision, TA-125-UB) was applied to each slide followed by 10 min of incubation at room temperature in a humid chamber. Kidney sections were incubated overnight at 4 °C with rabbit polyclonal endothelial nitric oxide synthase (eNOS, 1:100) and inducible nitric oxide synthase (iNOS, 1:100) antibodies (eNOS NeoMarkers; Ab iNOS Ab-1, Rabbit PAb, RB-1605-P, NeoMarkers Fremont, CA, USA), anti-tumor necrosis factor (TNF)-α (1:200) (rabbit polyclonal, Abcam ab66579, Abcam Cambridge, UK), and anti-interleukin (IL)-6 (1:200) antibodies (rabbit polyclonal, Abcam 6672, Abcam Cambridge, UK) and incubated for 1 h at room temperature with anti-myeloperoxidase (MPO) antibody (MPO rabbit RB-373-A, NeoMarkers Fremont, CA, USA) and anti-lipocalin-2 (NGAL) antibody (Abcam 41105, Abcam Cambridge, UK). The sections were washed in PBS three times for 5 min each time and then incubated for 30 min at room temperature with biotinylated goat anti-rabbit antibodies (LabVision, TP-125-BN). After the slides were washed in PBS, the streptavidin peroxidase label reagent (LabVision, TS-125-HR) was applied for 30 min at room temperature in a humid chamber. The colored product was developed by incubation with AEC. The slides were counterstained with Mayer’s hematoxylin (LabVision, TA-125-MH) and mounted in vision mount (LabVision, TA-060-UG) after being washed in distilled water. Both the intensities and distributions of iNOS and eNOS staining were scored. For each sample, a histological score (HSCORE) was derived by summing the percentage of cells that were stained at each intensity multiplied by the weighted intensity of the staining—HSCORE = S Pi (i + 1), where *i* is the intensity score, and Pi is the corresponding percentage of cells. The kidney sections were photographed using a Leica Qwin microscope.

As the measurement of cortical oxygenation and hemodynamic parameters were performed during the experiment, investigators were not blinded. However, creatinine levels, lactate and liver enzymes, and histological analysis were performed blinded.

### Statistical analysis

The results are expressed as the mean ± SEM. A normality test (D’Agostino test) was performed and, if data did not follow a gaussian distribution, a non-parametric test was used (Kruskal-Wallis test with a Dunn’s post hoc test). If data follow a gaussian distribution, statistical significance was calculated by one- and two-way analyses of variance (ANOVA) followed by Bonferroni post tests using GraphPad Prism (GraphPad Prism, Version 5, Software Program, San Diego, CA, USA). A *p* value <0.05 was considered statistically significant.

## Results

### Effect of fluid resuscitation and blood transfusion on systemic and renal hemodynamic parameters during sepsis

The MAP, renal blood flow and renal vascular resistance are provided in Table 1. Infusion of LPS induced early and persistent decreases in MAP and renal blood flow. FR significantly improved the MAP at T2 and T3 and the renal blood flow at T2. Similarly, BT significantly improved the MAP at T2 and T3 and the renal blood flow at T2. At the end of the experiment, there were no significant differences in terms of the systemic or renal hemodynamic parameters between the groups that received FR or BT after LPS infusion.

**Table 1:** Hemodynamic parameters

	Baseline (T0)	T1	T2	T3
Mean arterial pressure (mmHg)				
Control	91.5 ± 4.2	93 ± 4.1	94 ± 4.8	76.1 ± 2.3
LPS	88.7 ± 1.3	49 ± 5.1^**##**^	54.9 ± 6.2^**##**^	42.2 ± 4.4^**###**^
LPS + FR	90.8 ± 1.3	53.5 ± 3.2^**##**^	82.7 ± 2.1*	56.6 ± 2.1*^,##^
LPS + BT	89.5 ± 2.2	54.1 ± 1.7^**##**^	86.8 ± 1.7*	61.1 ± 3.2*
Heart rate (beats per minute)				
Control	232.9 ± 8.5	247.7 ± 7.7	248 ± 6.4	249.9 ± 13.4
LPS	236.3 ± 11.9	225.6 ± 5.2	226.9 ± 5.9	221 ± 8.5
LPS + FR	227.9 ± 8.7	219 ± 5.1	259 ± 7.7	258 ± 4.3
LPS + BT	255.3 ± 7.7	252.1 ± 5.6	258 ± 5.3	254.6 ± 6.5
Renal blood flow (ml/min)				
Control	7.5 ± 0.8	5.1 ± 0.2	5.2 ± 0.3	4.3 ± 0.2
LPS	7.1 ± 0.5	0.8 ± 0.3^**#**^	0.9 ± 0.3^**###**^	0.6 ± 0.3^**###**^
LPS + FR	6.3 ± 0.2	0.4 ± 0.1^**##**^	5.4 ± 0.9**	1.6 ± 0.3^**#**^
LPS + BT	6.6 ± 0.7	0.8 ± 0.3^**#**^	3.2 ± 0.4*	1.8 ± 0.3^**#**^
Renal vascular resistances (dynes·s·cm^–5^)				
Control	1291.9 ± 128.2	1823.2 ± 88.2	1844.4 ± 155.9	1784.3 ± 96.4
LPS	1275.2 ± 77.6	17,820 ± 7905.5	18,631.9 ± 11,394.9	20,237.7 ± 7939.2
LPS + FR	1449.9 ± 61.8	18,860.5 ± 6758.4	1853.3 ± 336.2	4390.5 ± 920.3
LPS + BT	1431.3 ± 124.1	12,133.9 ± 3493.6	2919.9 ± 388.8	4860.7 ± 1828.1

Hemodynamic parameters at four time points T0 (baseline), T1, T2, and T3 in the four groups. The data are expressed as the means ± SEMs. See Fig. 1 for definitions of the time points

^**#**^ *P* < 0.05, ^##^ *P* < 0.01, ^###^ *P* < 0.001 versus control; *P <0.05, **P < 0.01, versus LPS

*BT* blood transfusion, *FR* fluid resuscitation, *LPS* lipopolysaccharide

### Effects of fluid resuscitation and blood transfusion on renal microvascular oxygenation during sepsis

**Fig. 2:**
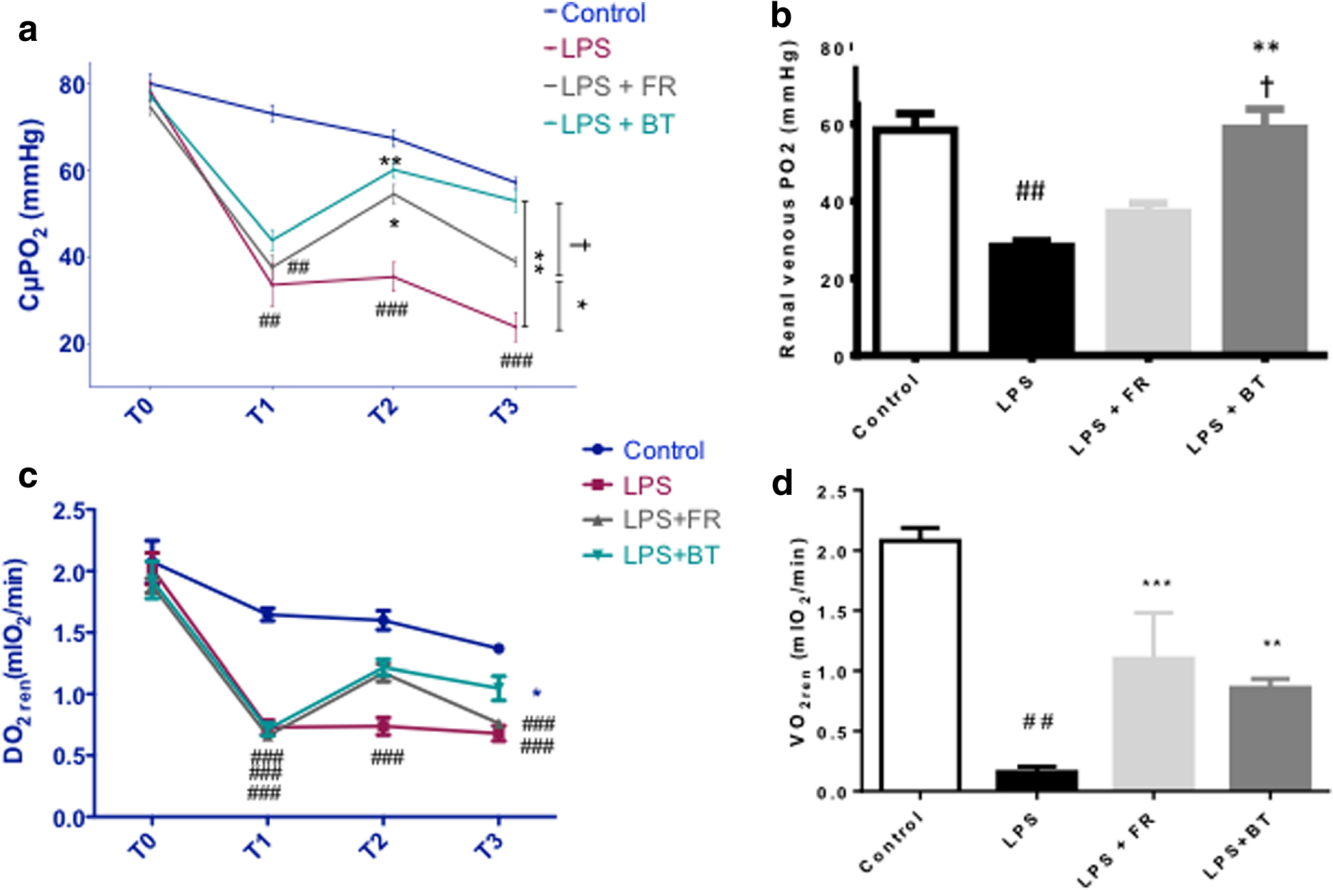
Renal oxygenation parameters. **a** Evolution of microvascular oxygen tension in the renal cortex (*CμPO*_*2*_) in the four groups at baseline (T0), T1, T2, and T3. **b** Renal venous partial pressure of oxygen (*PO*_*2*_) at T3 in the four groups. **c** Evolution of renal oxygen delivery (*DO*_*2ren*_) in the four groups at T0, T1, T2, and T3. **d** Renal oxygen consumption (VO_2ren_) at T3 in the four groups. ^##^ *P* < 0.01, ^###^ *P* < 0.001, versus control; *P < 0.05, **P < 0.01, ***P < 0.001, versus LPS; ^†^ *P* <0.05, versus LPS + FR. The values are shown as mean ± SEM. See Fig.1 for definitions of the time points. *BT* blood transfusion, *FR* fluid resuscitation, *LPS* lipopolysaccharide

The CμPO_2_, rvPO_2_, DO_2ren_, and VO_2ren_ values are illustrated in Fig. 2. LPS induced significant decreases in CμPO_2_ and rvPO_2_ from baseline to T3 (23.9 ± 8.9 mmHg in the LPS group versus 57.2 ± 2.9 mmHg in the control group at T3, *p* < 0.001, and 28.3 ± 3.9 mmHg in the LPS group versus 58.4 ± 9.7 mmHg in the control group at T3, respectively, *p* < 0.01). FR significantly improved the CμPO_2_ at T2 (54.5 ± 5.8 mmHg versus 35.4 ± 8.8 mmHg in the LPS group, *p* < 0.05), but this value began to decrease from T2 to T3 (38.9 ± 2.8 mmHg in the FR group at T3). BT significantly improved the CμPO_2_ at T2 (60.1.5 ± 4.9 mmHg, *p* < 0.01 versus the LPS group) and T3 (52.9 ± 6.9 mmHg versus 23.9 ± 8.9 mmHg in the LPS group, *p* < 0.01). There was a significant difference in the CμPO_2_ values at T3 between the LPS + FR group and the LPS + BT group (*p* < 0.05; Fig. 2a). BT improved the rvPO_2_ (58.9 ± 13.3 mmHg versus 28.3 ± 3.9 mmHg in the LPS group, *p* < 0.01). The increase in rvPO_2_ induced by BT was significantly greater than the increase in rvPO_2_ that was induced by FR (*p* < 0.05; Fig. 2b). The DO_2ren_ was significantly decreased after LPS and was partially restored at T3 in the BT group (*p* < 0.05 at T3 versus LPS). Differences between DO_2ren_ in the BT group and the FR group at T3 did not reach statistical significance (Fig. 2c). There was a significant drop in VO_2ren_ in the LPS group compared to the controls (*p* < 0.01). FR and BT both restored the VO_2ren_ at T3 (*p* < 0.001 and *p* < 0.01, respectively, at T3).

### Effects of fluid resuscitation and blood transfusion on hemoglobin, pH, serum lactate, and liver function

**Fig. 3:**
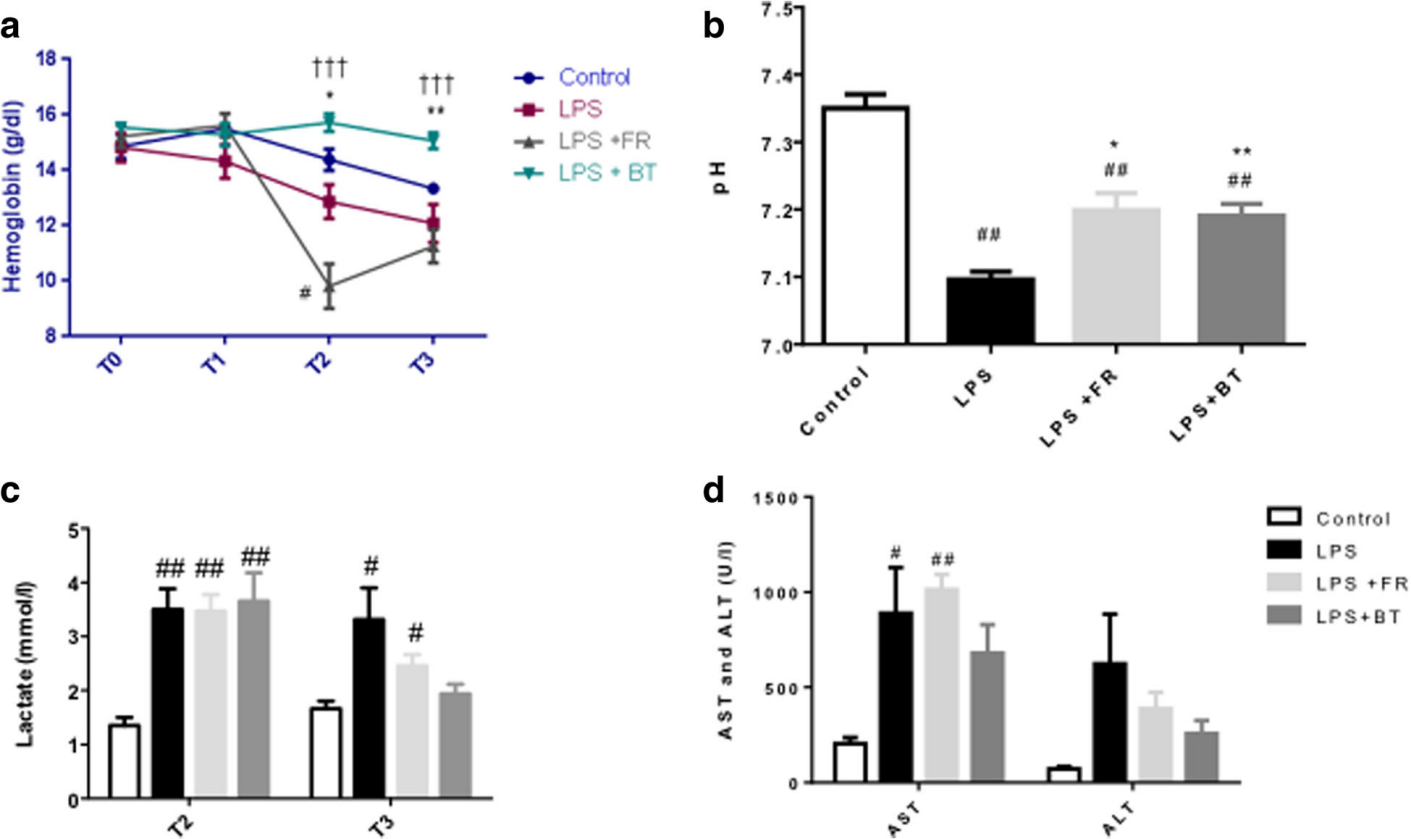
Hemoglobin, pH, serum lactate, and liver function during septic shock and resuscitation. **a** Hemoglobin concentrations at T0, T1, T2, and T3 in the four groups. **b** pH at T3 in the four groups. **c** Serum lactate levels at T2 and T3 in the four groups. **d** Aspartate aminotransferase (*AST*) and alanine aminotransferase (*ALT*) levels at T3 in the four groups. ^#^ *P* < 0.05, ^##^ *P* < 0.01, versus control; *P < 0.05, **P < 0.01, versus LPS; ^†††^ *P* <0.001, versus LPS + FR. The values are shown as mean ± SEM. See Fig.1 for definitions of the time points. *BT* blood transfusion, *FR* fluid resuscitation, *LPS* lipopolysaccharide

The hemoglobin levels, pH, serum lactate levels, and liver enzymes are illustrated in Fig. 3. As expected, FR induced significant decreases in hemoglobin values due to hemodilution (Fig. 3a). LPS infusion induced a significant drop in pH at T3 (*p* < 0.01 versus the control group) that was partially restored by FR and BT without any significant difference between the two groups (Fig. 3b). The serum lactate levels increased during sepsis (*p* < 0.01 at T2 and *p* < 0.05 at T3 in the LPS group versus the control group), but neither FR nor BT completely restored these values (Fig. 3c). Moreover, neither FR nor BT improved the LPS-induced increase in aspartate aminotransferase (AST), which is a surrogate marker for liver damage (Fig. 3d).

### Effects of fluid resuscitation and blood transfusion on renal function, metabolic cost, and tubular lesions during sepsis

**Fig 4:**
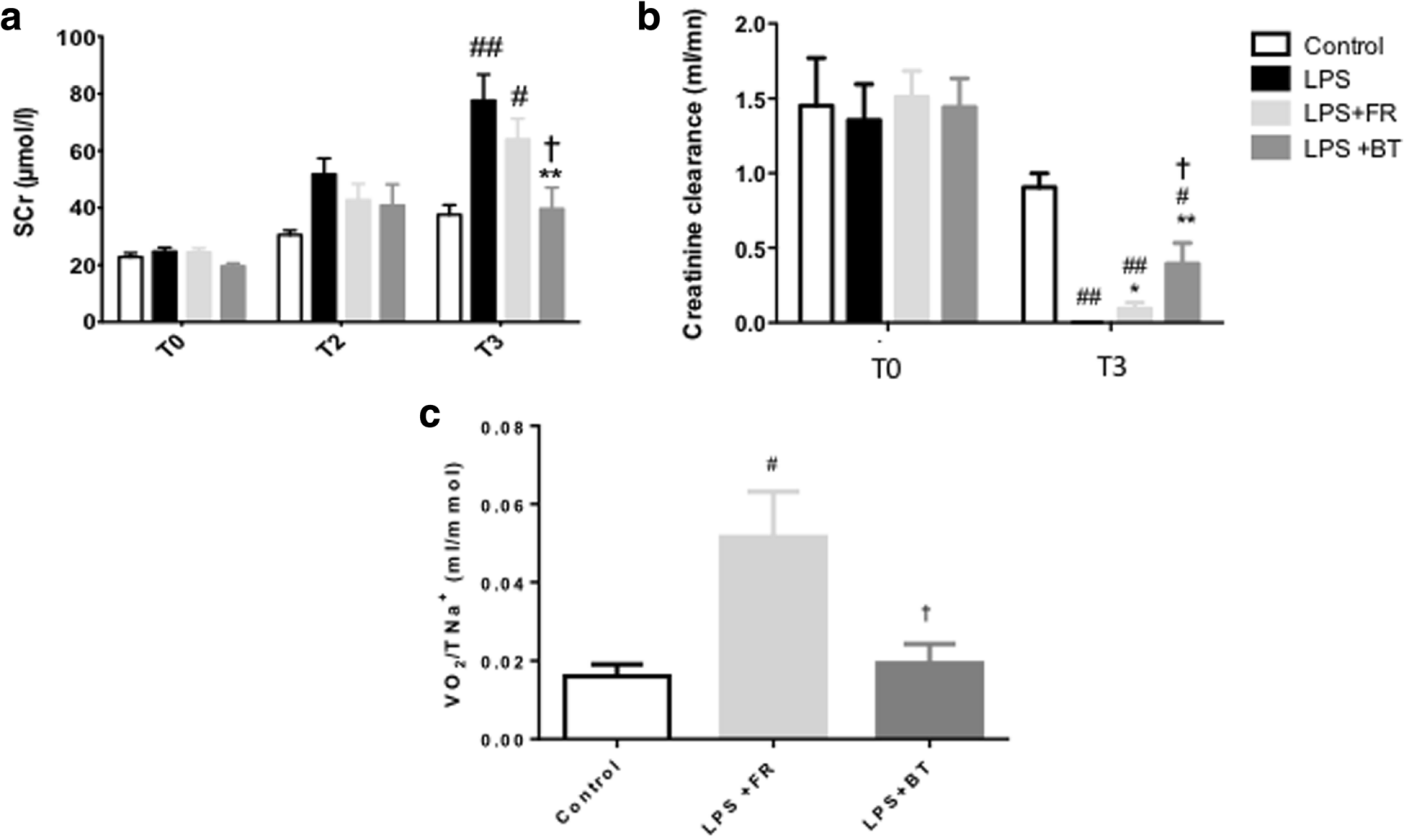
Kidney function and metabolic cost during septic shock and resuscitation. **a** Serum creatinine (*SCr*; μmol/l) at T0 and T3 in the four groups. **b** Creatinine clearance (ml/mn) at T0 and T3 in the four groups. **c** VO_2_/TNa^+^ (oxygen consumption per sodium reabsorbed (metabolic cost)) at T3 in the control group, LPS + FR group and LPS + BT group. ^#^ *P* < 0.05, ^##^ *P* < 0.01, versus control; *P < 0.05, **P < 0.01, versus LPS; ^†^ *P* <0.05, versus LPS + FR. The values are shown as mean ± SEM. See Fig.1 for definitions of the time points. *BT* blood transfusion, *FR* fluid resuscitation, *LPS* lipopolysaccharide

The creatinine levels are illustrated in Fig. 4a. At T1, all of the LPS-treated animals were anuric. This condition did not improve in the non-resuscitation group. All rats that received LPS suffered from AKI as reflected by increased plasmatic creatinine levels, and FR was not able to prevent AKI. The plasma creatinine levels did not differ significantly between the LPS group and the LPS + FR group. However, BT significantly decreased the plasma creatinine levels (39.6 ± 7.4 μmol/l in the LPS + BT group versus 77.7 ± 8.9 μmol/l in the LPS group at T3, *p* < 0.01, and 64 ± 7.2 μmol/l in the LPS + FR group, *p* < 0.05). Similarly, creatinine clearance significantly improved at T3 in the LPS + BT group (*p* < 0.05 versus LPS + FR group) (Fig. 4b).

The metabolic cost of sodium reabsorption as assessed with the VO_2_/TNa^+^ could not be calculated in the LPS group due to the absence of urine output after LPS administration without resuscitation. However, whereas LPS + FR induced an increase in VO_2_/TNa^+^ (*p* < 0.05 compared to the controls), the VO_2_/TNa^+^ ratio was not different after LPS + BT compared with the controls (*p* < 0.05 versus the FR group; Fig. 4c).

**Fig. 5:**
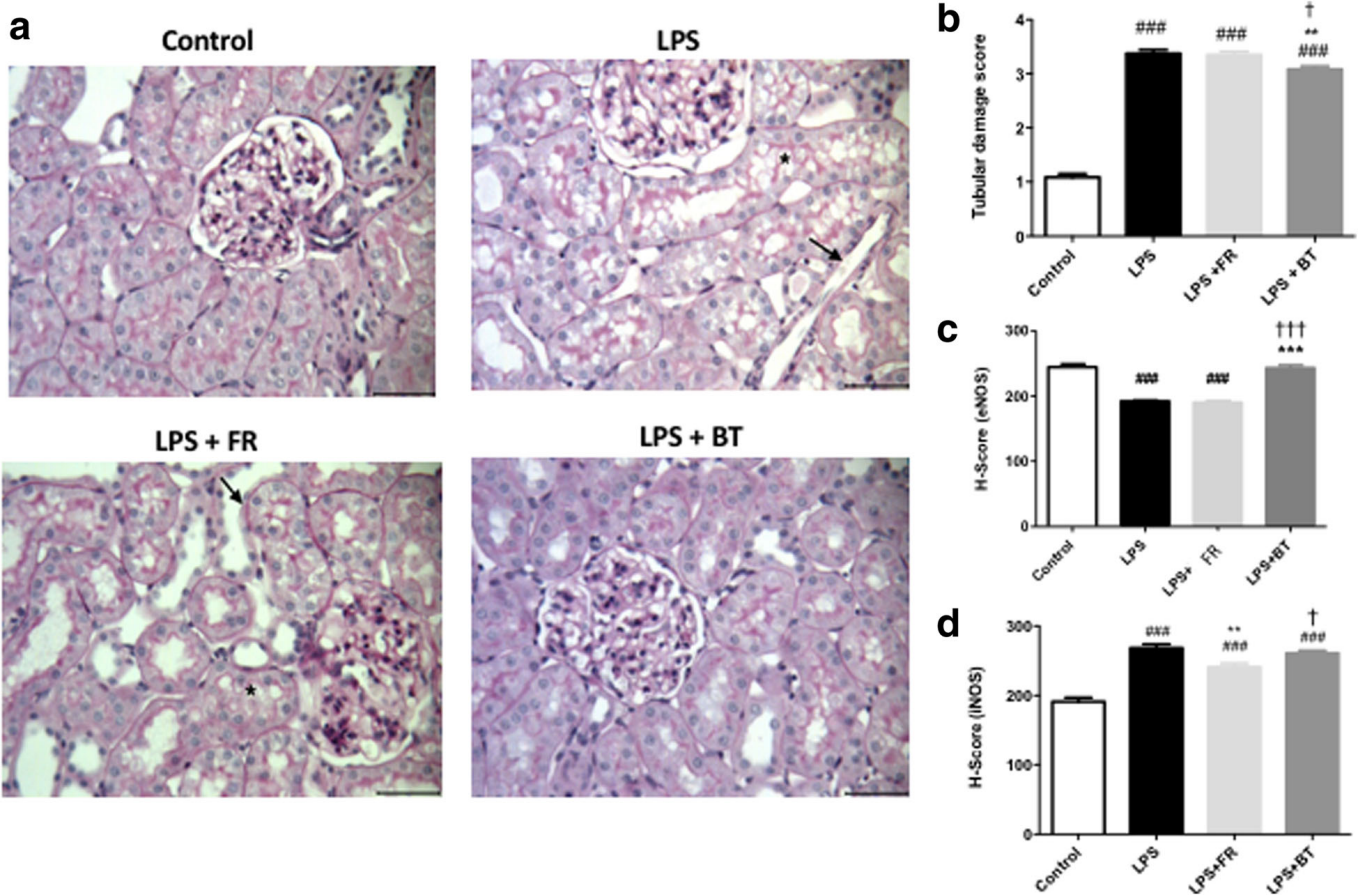
Renal histological changes and renal expressions of eNOS and iNOS during septic shock and resuscitation. **a** Representative periodic acid Schiff staining in the kidneys of the control, LPS, LPS + FR, and LPS + BT rats (original magnification × 400) and tubular injury scores in the four groups. Tubular vacuolization images of the LPS and LPS + FR groups further demonstrate the considerable tubular vacuolization (*asterisks*) as well as the loss of the brush border in the renal tubule (*closed arrows*). **b** Quantification of the tubular injury scores in the kidney sections. **c** Quantification of endothelial nitric oxide synthase (*eNOS*) staining intensities (*H-score*) in the kidney sections. **d** Quantification of inducible nitric oxide synthase (*iNOS*) staining intensities (H-score) in the kidney sections. The values are shown as mean ± SEM. ^###^ *P* < 0.001, versus control; **P < 0.01, versus LPS; ^†^ *P* <0.05, ^†††^ *P* <0.001, versus LPS + FR. *BT* blood transfusion, *FR* fluid resuscitation, *LPS* lipopolysaccharide

LPS induced tubular damage, including tubular vacuolization, tubular invagination, brush border loss, and luminal cast formation. BT, but not FR, significantly improved the tubular damage (mean histological score of 3 ± 0.06 in the LPS + BT group versus 3.4 ± 0.06 in the LPS group, *p* < 0.01, and 3.3 ± 0.06 in the LPS + FR group, *p* < 0.05; Fig. 5a and b).

### Effects of fluid resuscitation and blood transfusion on renal iNOS, eNOS expression, and tissue inflammatory markers

The renal expression of eNOS was markedly suppressed under endotoxin challenge (*p* < 0.001; Fig. 5c). BT significantly increased eNOS expression after LPS (mean H-score of 243 ± 4 in the LPS + BT group versus 192.3 ± 1.5 in the LPS group, *p* < 0.001). The renal expression of eNOS did not increase after FR (mean H-score of 190.4 ± 2.6). In contrast, the renal expression of iNOS was markedly increased under endotoxin challenge (*p* < 0.01; Fig. 5d). Whereas FR decreased iNOS expression after LPS (*p* < 0.01 in the LPS + FR group compared to the LPS group), BT did not change iNOS expression after LPS.

Neutrophil gelatinase-associated lipocalin (NGAL), a marker of AKI and kidney inflammation, significantly improved after BT compared to the LPS group (*p* < 0.05). However, the immunostaining intensity of NGAL was not statistically different between the LPS + FR and LPS + BT groups (Additional file 1: Figure S1). Sepsis induced an increase in pro-inflammatory cytokines (TNF-α and IL-6) into the kidney, but there was no statistical difference between the LPS + FR and LPS + BT groups (Additional file 1: Figure S2). Similarly, MPO activity within the kidney, a marker of oxidative stress, was not different between the two groups (Additional file 1: Figure S1).

## Discussion

In the present study, we demonstrated that BT restored renal microcirculatory oxygenation and, by extension, kidney function in a rat model of endotoxemia. BT had beneficial effects on kidney microvascular oxygenation, metabolic costs, and AKI. Sepsis-induced eNOS deficiency and renal damage were corrected by BT but not by FR.

Johannes et al. previously demonstrated the appearance of cortical microcirculatory hypoxic areas in endotoxin-induced renal failure in the rat [3]. In the present study, we confirmed the drop in renal cortical oxygenation induced by LPS. A part of this anoxia was due to the drop of the renal blood flow during septic shock. Indeed, FR was only able to temporarily improve cortical oxygenation by improving the renal blood flow. However, BT had stronger and longer-lasting effects on renal microvascular oxygenation despite similar renal blood flows. Microcirculatory dysfunction may contribute to renal hypoxia even in the absence of frank renal hypoperfusion. Several studies using microcirculatory techniques have now questioned the significance of arterial renal blood flow [15] and have suggested that the renal microcirculation is the hemodynamic culprit in the pathophysiology of septic AKI [16]. The microcirculation of the renal cortex has been demonstrated to be severely injured in animal models of sepsis [3, 5]. Legrand et al. demonstrated that the prevention of renal macrovascular hypoperfusion by FR cannot fully prevent renal microcirculatory oxygenation and perfusion dysfunction after LPS infusion in rats, despite a normalized renal blood flow [5]. Severe sepsis is characterized by a reduction in functional capillary density and an increase in blood flow heterogeneity. Indeed, the ischemic component is not found in global renal arterial blood flow but rather in a defect in the distribution of renal cortex microcirculation involving patchy areas of micro-ischemia [5]. Our study was not designed to demonstrate the distribution of red blood cells inside the kidneys after BT, and we cannot rule out the possibility that some areas inside the kidneys did not receive the oxygen delivered by the erythrocytes. However, the global effect of BT was an improvement in microvascular oxygenation that was independent of renal macrovascular perfusion. Moreover, the VO_2_/TNa^+^, which is a functional index of the efficiency of oxygen utilization for TNa^+^ and a functional parameter that reflects tubular injury [17], demonstrated more efficiency in oxygen utilization for tubular transport following BT than following FR. This improvement in kidney oxygenation participated in the improvement in kidney function. Indeed, renal tissue hypoxia is an important common feature of AKI [18] and is a major driver of the cascade of events that leads to cellular injury and vascular and tubular dysfunction [19]. In our study, improvements in kidney oxygenation translated into better renal outcomes as assessed by the serum creatinine levels and tubular damage.

These effects could be mediated in part by a restoration of eNOS activity after BT. Indeed, in accordance with previous studies we found a significant decrease in eNOS expression in the kidney under endotoxin challenge. In a murine model of cecal ligation and puncture-induced septic shock, Coletta et al. demonstrated that the lack of eNOS production alone may be sufficient to markedly exacerbate the severity of septic shock [20]. Nitric oxide (NO) is an important mediator of microvascular patency and blood flow, and strategies that aim to enhance endothelial eNOS activity have been found to decrease sepsis-induced neutrophil-endothelial cell interactions and may play a role in maintaining microvascular patency in septic shock [21]. Notably, Souza et al. reported similar findings that increasing hemoglobin levels using erythropoeitin in septic rats preserves renal eNOS expression and thereby prevents sepsis-induced AKI [22]. Thus, we hypothesize that the specific effect of BT on eNOS expression in the kidney may have improved the endothelial dysfunction and renal microcirculation in our model.

However, there are contradictory results from clinical studies in which BT was administered during sepsis. BT has been associated with increased mortality in subgroups of critically ill patients in both cohort studies and randomized trials [23, 24], but there have also been cohort studies in which transfusion was associated with improved survival, including in patients with sepsis [25, 26]. In a recent randomized controlled study, Holst et al. found that mortality rates were similar in patients who received BT at a higher hemoglobin threshold and those who received BT at a lower threshold during septic shock [27]. Our study did not investigate long-term survival or outcomes. We measured serum lactate as a global perfusion biomarker and liver enzymes as markers of liver function. In accordance with the study of Holst et al., we did not find any benefit of BT on liver function or lactic acidosis when compared with FR. How then can we explain the specific protective effect of BT on renal function? First, some data suggest a direct influence of hemodilution on microvascular flow and renal oxygen supply. The critical hematocrit associated with a decrease in microvascular PO_2_ has already been found to be much higher for the kidney than for the intestines and the heart [28]. The benefit of increasing the hematocrit level with BT may therefore be greater for the kidney than for other organs. Second, there is accumulating evidence that endothelial cell phenotypes vary between different organs and exhibit remarkable heterogeneity in structure and function [29, 30]. Under similar conditions, eNOS activity is also highly variable between organs [30, 31]. eNOS is highly expressed in glomerular endothelial cells and the endothelium of cortical vessels in control and diseased kidneys [32]. Thus, we hypothesize that, depending on the level of eNOS activity and the interaction between BT and endothelial cells, the influence of BT may vary greatly from one organ to another.

When studying the effect of BT during sepsis, another important issue needs to be accounted for: the failure to identify significant improvements in outcomes in clinical studies may pertain to the potentially deleterious effect of blood storage. In our study, we transfused fresh blood to the septic rats. During storage, erythrocytes are exposed to and produce substances that impair their function when they are returned to the circulation [33]. In this situation, BT may, rather than improving oxygenation, worsen oxygen balance and AKI. The use of fresh blood in our study may therefore, in part, explain the beneficial effects we observed in terms of renal oxygenation and AKI. However, a recent randomized trial comparing transfusion of fresh red cells to standard-issue red cells did not found any difference in terms of mortality in a large population of critically ill patients [34].

### Limitations

Our study has several limitations. First, the acute and lethal model of endotoxic shock (in a limited sample size of animals) used herein is clearly not fully representative of human septic shock. LPS causes much earlier and higher peak levels of cytokine expression compared with levels observed in human sepsis. However, endotoxemic challenge with LPS remains a useful tool for interrogating a simpler subset of the complex trajectory of sepsis. Second, our rats were resuscitated with fluids but not norepinephrine. The use of norepinephrine has been shown to be associated with better tissue oxygenation when compared with fluid resuscitation alone [35, 36].

Finally, the modalities, timing, and threshold of hemoglobin that need to be targeted have to be defined before conducting a clinical trial focusing on sepsis-induced AKI.

## Conclusion

In conclusion, BT might be useful as a renal protective strategy to preserve renal oxygenation and kidney function during sepsis. However, additional studies are warranted to evaluate the true clinical value of BT in this setting and its prolonged beneficial effects on kidney function.

## Additional files

Additional file 1: Surgical preparation and blood gas measurements are detailed in the supplemental data. **Figure S1.** Myeloperoxydase (*MPO*) immunostaining and neutrophil gelatinase-associated lipocalin (*NGAL*) immunostaining in kidney sections in the four groups. ***P* < 0.01. **Figure S2.** Immunostaining of tumor necrosis factor alpha (*TNF-α*) and interleukin-6 (*IL-6*) in kidney sections in the four groups. ^#^ *P* < 0.05, ^###^ *P* < 0.001, versus control; **P* < 0.05, versus LPS. (DOCX 103 kb)

## Abbreviations

AKI: Acute kidney injury
AST: Aspartate aminotransferase
BT: Blood transfusion
CμPO_2_: Microvascular oxygen tension in the renal cortex
DO_2ren_: Renal oxygen delivery
eNOS: Endothelial nitric oxide synthase
FR: Fluid resuscitation
IL: Interleukin
iNOS: Inducible nitric oxide synthase
LPS: Lipopolysaccharide
MAP: Mean arterial pressure
MPO: Myeloperoxidase
NGAL: Neutrophil gelatinase-associated lipocalin
NO: Nitric oxide
PBS: Phosphate-buffered saline
PO_2_: Partial pressure of oxygen
rvPO_2_: Renal venous partial pressure of oxygen
TNF: Tumor necrosis factor
VO_2ren_: Renal oxygen consumption
