# Supplementary material for: Blood transfusion improves renal oxygenation and renal function in sepsis-induced acute kidney injury in rats
Source: Crit Care. 2016 Dec 20;20:406. doi: 10.1186/s13054-016-1581-1 (PMC5168817; doi:10.1186/s13054-016-1581-1)
Supplement: Additional file 1: — Surgical preparation and blood gas measurements are detailed in the supplemental data. Figure S1. Myeloperoxydase (MPO) immunostaining and neutrophil gelatinase-associated lipocalin (NGAL) immunostaining in kidney sections in the four groups. **P < 0.01. Figure S2. Immunostaining of tumor necrosis factor alpha (TNF-α) and interleukin-6 (IL-6) in kidney sections in the four groups. # P < 0.05, ### P < 0.001, versus control; *P < 0.05, versus LPS. (DOCX 103 kb) [file 13054_2016_1581_MOESM1_ESM.docx]

**Supplemental data**

**Methods**

**Surgical preparation**

All animals were anesthetized with an intraperitoneal injection of a mixture of 90 mg/kg ketamine (Nimatek^®^, Eurovet, Bladel, The Netherlands), 0.5 mg/kg dexmedetomidine (Dexdomitor, Pfizer Animal Health BV, Capelle aan den IJssel, The Netherlands), and 0.05 mg/kg atropine-sulfate (Centrafarm Pharmaceuticals BV, Etten-Leur, The Netherlands). After performing a tracheotomy, the animals were mechanically ventilated with a fraction of inspired oxygen (FiO_2_) of 0.4. Body temperature was maintained at 37±0.5 °C during the entire experiment with an external thermal heating pad. The ventilator settings were adjusted to maintain an arterial partial pressure of carbon dioxide (pCO_2_) between 35 and 40 mmHg. For drug and fluid administration and hemodynamic monitoring, the vessels were cannulated with polyethylene catheters with an outer diameter of 0.9 mm (Braun, Melsungen, Germany). A catheter in the right carotid artery was connected to a pressure transducer to monitor the mean arterial blood pressure (MAP) and heart rate. The right jugular vein was cannulated for continuous infusion of 50 mg/kg/h ketamine dissolved in saline at a rate of 15 mL/kg/hour for maintenance of the intravascular volume and anesthesia. The right femoral artery was cannulated to draw drawing blood samples, and the right femoral vein was cannulated for drug administration. The left kidney was exposed and immobilized in a Lucite kidney cup (K. Effenberger, Pfaffingen, Germany) via an ~4-cm incision in the left flank in each animal. The renal vessels were carefully separated while preserving the nerves and the adrenal gland. A perivascular ultrasonic transient time flow probe was placed around the left renal artery (type 0.7 RB Transonic Systems Inc., Ithaca, NY, USA) and connected to a flow meter (T206, Transonic Systems Inc., Ithaca, NY, USA) to continuously measure renal blood flow (RBF). The left ureter was isolated, ligated, and cannulated with a polyethylene catheter for urine collection. After the surgical preparation, one optical fiber was placed 1 mm above the decapsulated kidney to measure the renal microvascular and oxygenation using phosphorimetry. The surgical field was covered with a humidified gauze compress throughout the entire experiment to prevent the drying of the exposed tissues.

The changes in the oxygen-dependent quenching of palladium-porphyrin (Pd-porphyrin) phosphorescence (1) in the renal cortex were measured with an optical fiber that was positioned a few millimeters above the kidney, and the values were converted into local microvascular PO2 values as previously described (2-3).

***Blood gas measurements***

After the infusion of the palladium-porphyrin (Pd-porphyrin) solution and 45 min of stabilization, a baseline blood sample (0.3 ml) was collected from the femoral artery (T0), and additional blood samples were collected at 120 min after LPS infusion (T1), 15 min after resuscitation (early resuscitation phase, T2), and 180 min after resuscitation (late resuscitation phase, T3). The blood samples were replaced by the same volume of balanced colloid solution. Venous blood was obtained from the renal vein at the end of experiment. The samples were used to determine the blood gas parameters (Radiometer ABL 505 Blood Gas Analyzer, Copenhagen, Denmark.

**Figure S1**


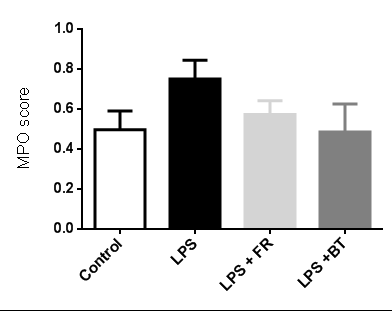

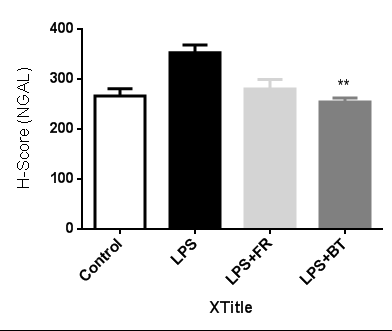


Figure Legend: Myeloperoxydase immunostaining and Neutrophil Gelatinase Associated Lipocalin (NGAL) immunostaining in kidney sections in the four groups ; ** p<0.01

**Figure S2**

**** ****

Figure legend: Immunostaining of Tumor Necrosis Factor-α (TNF-α) and Interleukin-6 (IL-6) in kidney sections in the four groups. ### p<0.001 versus control ; # p<0.05 versus control; * p<0.05 versus LPSα

1. Vanderkooi JM, Maniara G, Green TJ, et al. An optical method for measurement of dioxygen concentration based upon quenching of phosphorescence. J Biol Chem 1987;262(12):5476-5482.

2. Rubin O, Crettaz D, Tissot JD, et al. Microparticles in stored red blood cells: submicron clotting bombs? Blood Transfus 2010;8 Suppl 3:s31-38.

3. Sinaasappel M, Donkersloot C, van Bommel J, et al. PO2 measurements in the rat intestinal microcirculation. Am J Physiol 1999;276(6 Pt 1):G1515-1520.
